# Supplementary material for: Novel Anterior Cranial Base Area for Voxel-Based Superimposition of Craniofacial CBCTs
Source: J Clin Med. 2022 Jun 20;11(12):3536. doi: 10.3390/jcm11123536 (PMC9225157; doi:10.3390/jcm11123536)
Supplement: Supplementary file 1 [file jcm-11-03536-s001.zip › jcm-1739829-supplementary.pdf]

Novel Anterior Cranial Base Area for Voxel-Based Superimposition of Craniofacial CBCTs

Georgios Kanavakis, Mohammed Ghamri, and Nikolaos Gkantidis

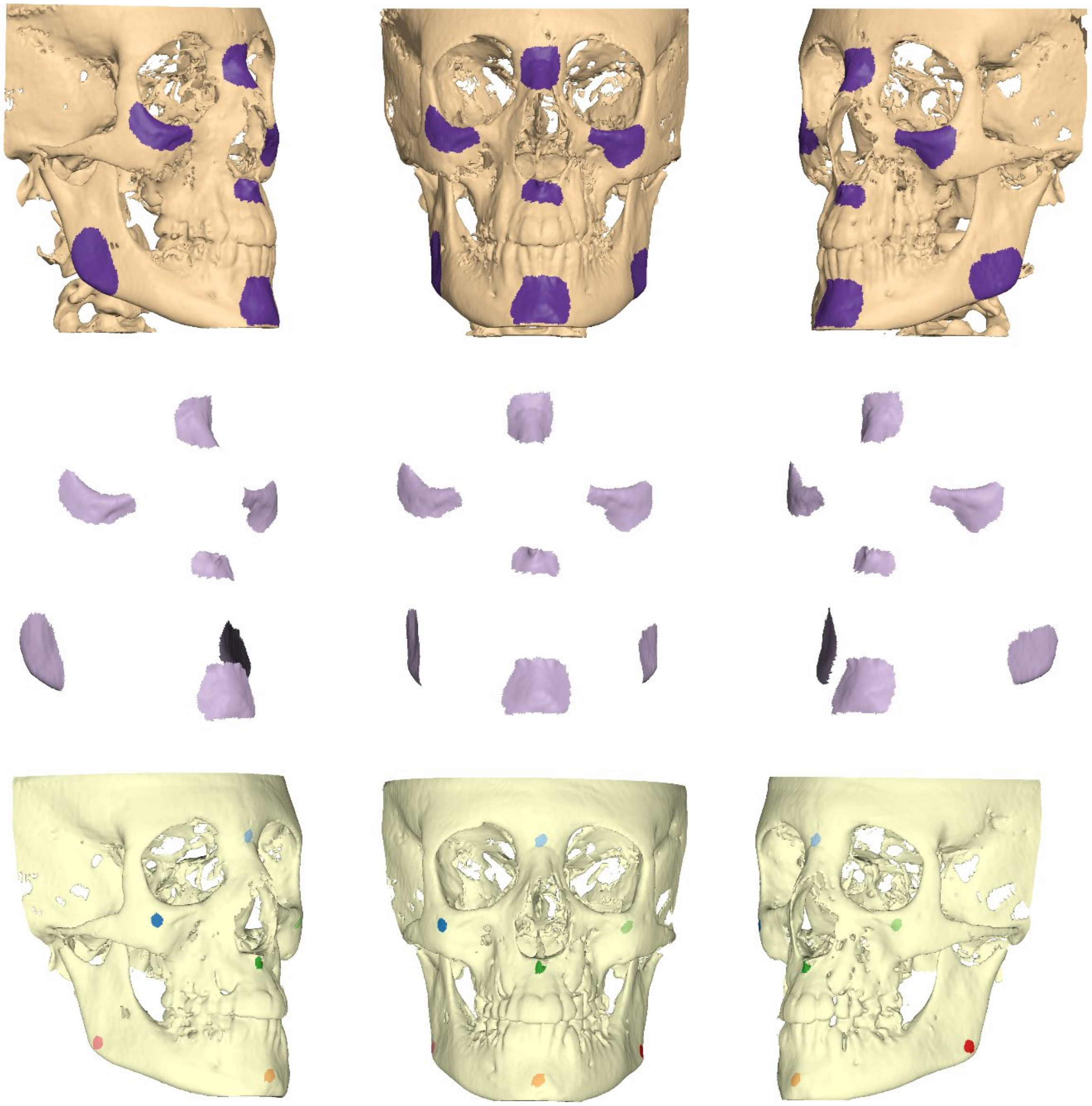

Supplementary Figure S1. Manual extraction of the outer surface layer of the T1 models to avoid bias in measurements. The upper and middle rows depict the areas extracted from each T1 surface model (outer surface layer) to enable the unbiased calculation of distances between the seven measurement areas selected on the T0 models (lower row) and the corresponding areas extracted from the T1 surface models.

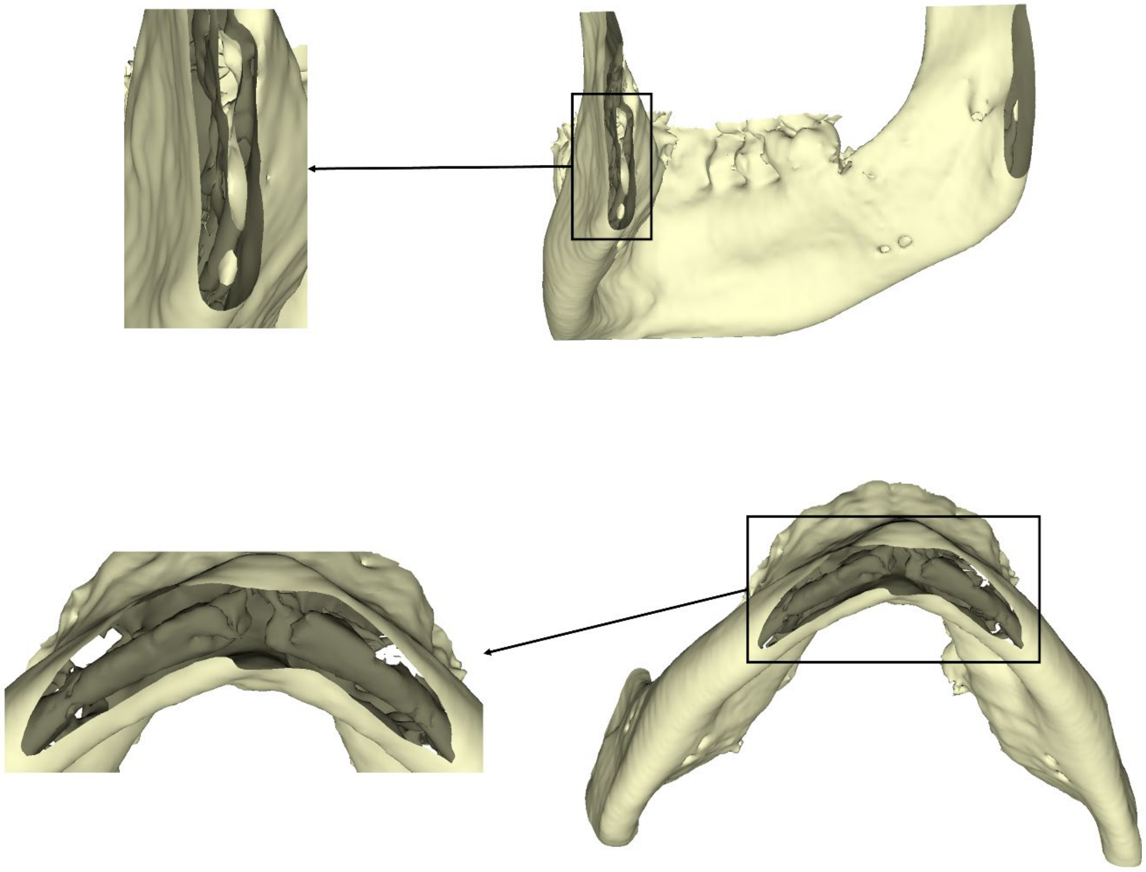

Supplementary Figure S2. Cross-sections of 3D surface models showing the intermediate inner structures that could form during bone segmentation from the original radiographic volumes and bias the calculations of distances between the outer surfaces of the T1 models and the corresponding seven measurement areas selected on the T0 models.
